# Generation of the NIR Spectral Band for Satellite Images with Convolutional Neural Networks

**DOI:** 10.3390/s21165646

**Published:** 2021-08-21

**Authors:** Svetlana Illarionova, Dmitrii Shadrin, Alexey Trekin, Vladimir Ignatiev, Ivan Oseledets

**Affiliations:** 1 Skolkovo Institute of Science and Technology, 143026 Moscow, Russia; Dmitry.Shadrin@skolkovotech.ru (D.S.); A.Trekin@skoltech.ru (A.T.); V.Ignatiev@skoltech.ru (V.I.); i.oseledets@skoltech.ru (I.O.); 2 Institute of Numerical Mathematics of Russian Academy of Sciences, 119333 Moscow, Russia

**Keywords:** GAN, satellite imagery, convolutional neural network, near-infrared channel, feature engineering

## Abstract

The near-infrared (NIR) spectral range (from 780 to 2500 nm) of the multispectral remote sensing imagery provides vital information for landcover classification, especially concerning vegetation assessment. Despite the usefulness of NIR, it does not always accomplish common RGB. Modern achievements in image processing via deep neural networks make it possible to generate artificial spectral information, for example, to solve the image colorization problem. In this research, we aim to investigate whether this approach can produce not only visually similar images but also an artificial spectral band that can improve the performance of computer vision algorithms for solving remote sensing tasks. We study the use of a generative adversarial network (GAN) approach in the task of the NIR band generation using only RGB channels of high-resolution satellite imagery. We evaluate the impact of a generated channel on the model performance to solve the forest segmentation task. Our results show an increase in model accuracy when using generated NIR compared to the baseline model, which uses only RGB (0.947 and 0.914 F1-scores, respectively). The presented study shows the advantages of generating the extra band such as the opportunity to reduce the required amount of labeled data.

## 1. Introduction

Machine learning techniques allow researchers to achieve high performance in a wide range of remote sensing tasks by leveraging spectral bands of different wavelengths [1]. One essential spectrum interval for the remote sensing image analysis is represented by the near-infrared (NIR) channel. The classical approaches in landcover classification tasks often use NIR-based spectral indices such as the Normalized Difference Vegetation Index (NDVI) or the Enhanced Vegetation Index (EVI) to assess the vegetation state [2]. This spectral band is widely used in many applications, including forestry [3,4], agriculture [5,6], and general landcover classification [7,8]. However, there are still cases when the NIR band is not presented in the available data [9,10]. Thus, the researchers rely only on RGB. For example, the Maxar Open Data Program [11] provides only RGB images. Many aerial imaging systems are also limited to visible wavelength ranges.

**Figure 1:**
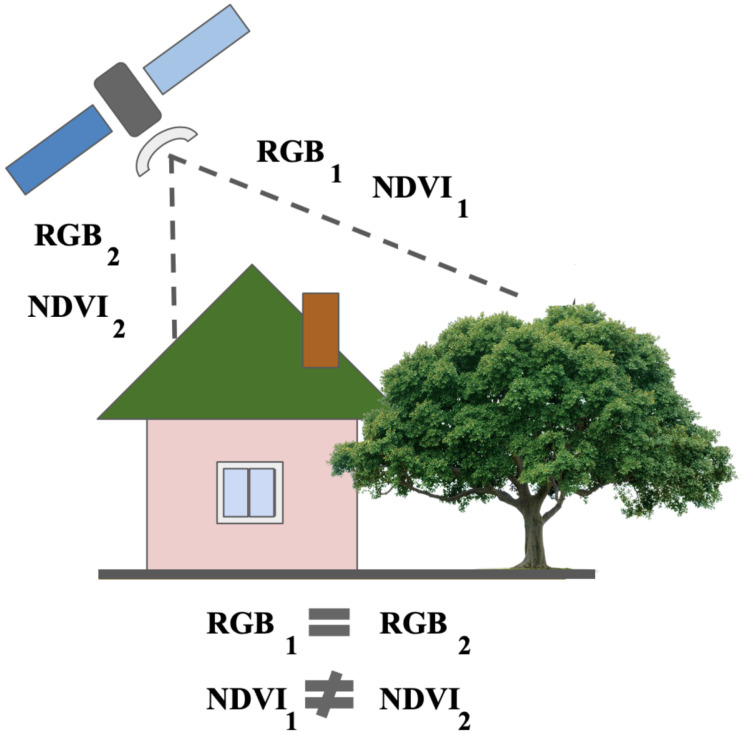
Objects with the same spectral values in the RGB range can belong to significantly different classes. For these objects, spectral values beyond the visible range differ. These differences can be illustrated using vegetation indices such as the NDVI in the case of an artificial object and a plant during the vegetation period.

The NIR band cannot be extracted from RGB bands. A simple example is provided in Figure 1. For both the green tree and the green roof, the RGB values are the same. However, the values differ drastically in the NIR spectral range, as the metal roof does not have the vegetation properties that affect the NIR. On the other hand, indirect features can be used to evaluate the NIR value. In general, all roofs have a lower NIR values than any healthy tree during the vegetation period. Therefore, it is possible to make assumptions about the NIR value based on the object’s shape and texture. This study investigates how neural networks can be applied to solve the NIR generation task by learning the statistical distribution of a large unlabeled dataset of satellite images.

In [12], a similar problem of generating the NIR channel from RGB was described. The proposed solution was based on the K-Nearest Neighbor classification algorithm and was focused on the agricultural domain. The researchers show in [12] a high demand for the generated NIR data, which can solve particular problems. However, the neural network approach was beyond the scope of the present study for image generation. In [13], they generated synthetic spectral bands for archive satellite images using Landsat data. Synthetic satellite imagery generation from Sentinel-2 (with the spatial resolution more than 10 meters per pixel) was considered in [14,15]. However, in our work, we were focused on high-resolution satellite images as they provide valuable texture information.

Generative adversarial networks (GANs) have achieved great results in recent years [16]. The basis of this approach consists of two neural network models that are trained to beat each other. The first network (generator) aims to create instances as realistically as possible, and the second network (discriminator) learns to verify whether the instance is fake or real. Conditional GANs (cGAN) have proven to be a promising approach in various fields using additional conditions in the generation process. Conditional GANs were implemented to solve different tasks such as image colorization [17], including infrared input [18] and remote sensing data [19,20,21,22], and style transfer [23,24].

Pix2pix GAN, as described in [24], proposes an image-to-image translation approach. Previous studies have shown a lack of generalization for other problems. Authors of [24] aimed to develop an efficient framework that can be successfully implemented to solve a wide variety of tasks, such as image colorization, synthesizing images from a labeled map, generating land-cover maps from remote sensing images, changing the style, etc. Pix2pix GAN uses a “U-Net”-based architecture as a generator and a convolutional “PatchGAN” as a discriminator. The model was trained to estimate image originality separately for each small region. The authors used the following objective function $G^{*}=\arg\min_{G}\;\max_{D}L_{cGAN}\left(G,D\right)+\lambda L_{L1}\left(G\right)$ to train the model. The Pix2pix approach enhancements were provided by the authors of [25].

One prevalent computer vision task is image colorization, which is required to obtain color images from grayscale ones [26]. One of the earliest works using texture information for this task is [27]. In recent years, GANs (in particular cGANs) have become a popular approach for such a challenge, in particular, in the remote sensing domain [20,28]. In the image colorization task, cGANs take a condition that should be utilized for new image generation. The results for such a task can be evaluated visually. This challenge share similarities with the NIR generation problem. As an input, grayscale images are received, and as an output, an RGB image is created. In contrast, for NIR, we strive to obtain one channel from three channels. Unlike mapping grayscale to RGB, NIR does not include a mixture of RGB; NIR even lies in a distant wavelength region from RGB. It makes the task more challenging. Moreover, in the colorization problem, the choice of color sometimes depends on the statistical distribution in the training set (for example, the color of the car might depend on the number of cars for each color). Such mismatches in colorization might not be treated as a severe mistake, and it does not corrupt the sense of the natural source of objects or phenomena. In contrast, for NIR in vegetation tasks, there is a strong connection between chlorophyll content and the intensity of the channel value [29]. A neural network can extract structure features such as shape and texture characteristics. We attempt to combine them with RGB values to generate the NIR band artificially and save the physical sense of this channel as much as possible.

In the remote sensing domain, the opportunity to work with multiple satellite data simultaneously is essential in various cases [30]. In [31], the authors consider WorldView and Planet imagery. WorldView has a higher spatial resolution, while Planet has a higher temporal resolution. Therefore, by combining these data, researchers can solve particular problems rapidly and with better quality. In [32], Modis and Landsat images fusion was considered in the flood mapping case. In [33], they combine images from WorldView2, Rapid Eye, and PlanetScope platforms to solve the forest degradation problem. When images from several sensors were available, the highest spatial resolution images were always preferred. Therefore, in the remote sensing domain, acquisition dates can vary for different satellites, and for monitoring, it is crucial to work with all available data sources. However, when a computer vision model uses data from different distributions, it can decrease prediction quality. One of the objectives of our study was to examine the importance of the NIR band for cross-domain stability.

**Figure 2:**
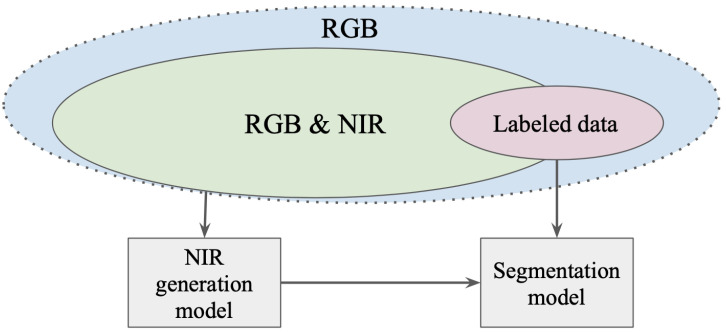
A large amount of RGB & NIR data without markup that can be further leveraged in semantic segmentation tasks when NIR is not available in some particular cases.

In our study, we examine whether the cGAN image generation approach can produce sufficient results for image segmentation purposes. Multiscale contextual features and spatial details are highly important in the remote sensing domain [34]. Therefore, we aim to apply the NIR generation as a feature-engineering method, creating a new feature (NIR reflectance) that is not present in the original feature space (RGB reflectance). We also study original and artificially generated NIR in the cross-domain stability problem, as convolutional neural network (CNN) robustness for various data is vital in the remote sensing domain [35]. We aim to use a vast amount of RGB & NIR data without markup that can be further leveraged in semantic segmentation tasks when NIR is not always available Figure 2.

We propose and validate an efficient approach to produce an artificial NIR band from the RGB satellite image. A state-of-the-art Pix2pix GAN technique is implemented for this task and compared with a common CNN-based approach for the regression task. WorldView-2 high-resolution data are leveraged to conduct image translation from RGB to NIR with further verification on PlanetScope and Spot-5 RGB images. We also investigate how original and artificially generated NIR bands affect both CNN and Random Forest (RF) [36] predictions in forest segmentation tasks compared to only RGB data. The experiments involve two significant practical cases: two data source combinations (PlanetScope and Spot-5) and different amount of labeled training data (the total dataset size for the segmentation task is 500.000 hectares). The contribution of the presented work is as follows:

- We propose the approach for feature-engineering based on the NIR channel generation via cGANs.
- We investigate the impact of artificially generated and real NIR data on the model performance in the satellite image segmentation task. We also examine the NIR channel contribution in reducing labeled dataset size with minimum quality loss. The NIR channel for satellite cross-domain stability is considered.

## 2. Materials and Methods

### 2.1. Dataset

We leveraged WorldView-2 satellite imagery downloaded from GBDX [37] to train the generative models. For forest segmentation experiments, we used the satellite data provided by the SPOT-5 [38] satellite and the PlanetScope [39] satellite group. The imagery has a high spatial resolution of 2–3 meters per pixel in four spectral channels (red, green, blue, near-infrared). All images were georeferenced and had values equal to the surface reflectance. Overall, two datasets were used in this work:

The first dataset used in this work was for cGAN model training. The dataset consists of RGB and NIR channels from the same satellite (WorldView-2). It covers different regions of Russia and Kazakhstan with approximately the same climate and ecological conditions. The total territory is about 900,000 ha. The datasets consist of varying land cover classes such as crops, forests, non-cultivated fields, and human-made objects. Images with dates from May to September were chosen to represent the high-vegetation period.

The second dataset was used to test the real and artificial NIR channel’s influence compared to the bare RGB image. This dataset includes PlanetScope and Spot-5 imagery. The resolution of images ranges between 2 and 3 meters, depending on the view angle. The markup for the study region consists of the binary masks of the forested areas and other classes in equal proportion, covering 500,000 ha. The labeled markup was used for the binary image segmentation problem. The region was split into test and train parts in the proportion of 0.25:0.75.

### 2.2. Artificial NIR Channel Generation

**Figure 3:**
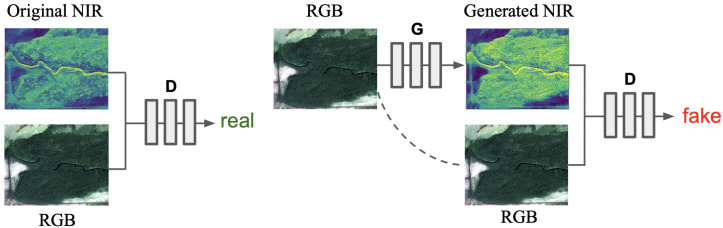
Training procedure for GAN using the RGB image as an input and the NIR band as a condition.

To generate the NIR band from RGB, we used cGAN. We chose the Pix2pix approach for this task because it performs quite well for image translation problems [40,41]. For the generator, we used the U-Net [42] architecture with the Resnet-34 [43] encoder. For the discriminator, the PatchGAN as described in [24] with various receptive field sizes was used. The training procedure is shown in Figure 3. There were two models: the generator and the discriminator. The generator was trained to create artificial NIR images, using the RGB image as a conditional input. The discriminator received an RGB image in combination with the alleged NIR image. Then, there were few possible scenarios: (1) the NIR was original, and the discriminator succeeded in ascertaining it; (2) the NIR was fake, but the discriminator failed by treating it as original; (3) the NIR was original, but the discriminator mistook for fake; (4) the NIR was fake, and the discriminator exposed it. Although this model was trained simultaneously, we ultimately strove to receive a high performing generative model, to solve the objective of the study. For further analysis, only the generator was considered. Unlike classical machine learning techniques, which usually work only with one particular point (see [12]), the U-Net generator processes a particular neighborhood and learns how to summarize 3-dimensional information.

We compared the cGAN-based approach with the simple CNN-based one where U-Net with Resnet-34 encoder was trained to solve the regression problem.

We considered the root mean square error (RMSE), mean absolute error (MAE), and mean bias error (MBE) for the model’s performance evaluation as follows:

(1) $$\begin{array}{c} RMSE=\sqrt{\frac{\sum_{i=1}^{n}{\left(y_{i}-\hat{y_{i}}\right)}^{2}}{n}} \end{array}$$

(2) $$\begin{array}{c} MAE=\frac{\sum_{i=1}^{n}\left|y_{i}-\hat{y_{i}}\right|}{n} \end{array}$$

(3) $$\begin{array}{c} MBE=\frac{\sum_{i=1}^{n}\left(y_{i}-\hat{y_{i}}\right)}{n} \end{array}$$

where $\bar{y}$ is the mean target value among all pixels, $\hat{y_{i}}$ is the predicted value of the *i*th pixel, $y_{i}$ is the target value of the *i*th pixel, and *n* is the pixel number.

### 2.3. Forest Segmentation Task

To empirically evaluate the usefulness of the original and artificially generated NIR channel to solve real image segmentation problems, we considered the forest segmentation task with high-resolution satellite imagery. In this task, a CNN model was trained to ascribe each pixel with the forest content label.

We used the common solution for the image semantic segmentation: U-Net [42] with the ResNet-34 [44] encoder. The chosen architecture is widely implemented in the remote sensing domain [45]. We conducted experiments with different input channels: only RGB, RGB & original NIR, and RGB & generated NIR. The model output was a binary mask of the forest landcover, which was evaluated against the ground truth with an F1-score. We also assessed the original and artificially generated NIR in the same task using classical machine learning approach. We trained a Random Forest (RF) classifier [36]. The RF implementation was from [46] with the default parameters the same as in [36]. Each pixel was considered as an object for the classification.

(4) $$\begin{array}{c} precision=\frac{TP}{TP+FP},\\ recall=\frac{TP}{TP+FN},\\ F1=\frac{2*precision*recall}{precision+recall} \end{array}$$

where TP is the True Positive (the number of correctly classified pixels of the given class), FP is the False Positive (the number of pixels classified as the given class while, in fact, being of the other class, and FN is the False Negative (the number of pixels of the given class, that were missed by the method).

### 2.4. NIR Channel Usage

We conducted an experiment that estimated the dependency of the segmentation quality on the training dataset size in both RGB and RGB & NIR cases. We randomly split and chose 50% and 30% of the initial training dataset (test data were the same for these random splits). The same experiment was repeated both for the SPOT and Planet imagery but separately for each data source.

**Figure 4:**
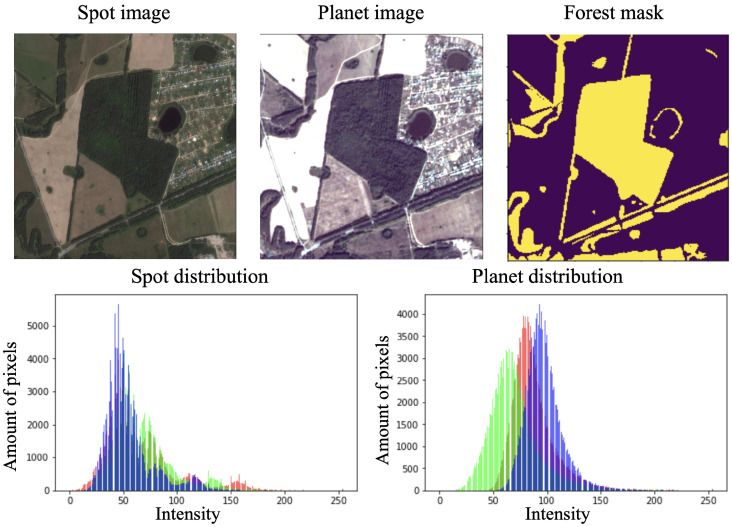
Original SPOT and Planet images (without any enhancements) and their RGB spectral values distribution. The histograms were computed within the forest area. Although the presented images are from the summer period, their spectral values differ drastically, as the histogram shows.

In the second study, we considered data from different sources (both PlanetScope and SPOT data) simultaneously. Even if we have two images of the same date, region, and resolution but from various providers, sensors systems and image preprocessing can make them radically different from each other. The intensity distribution for images from Spot and Planet are shown in Figure 4. Such differences can be crucial for machine vision algorithms and lead to a reduction in prediction quality. Therefore, it can be treated as a case of a more complex multi-domain satellite segmentation task. To estimate the importance of the original and artificial NIR channels for different satellite data, we conducted the following experiment. The CNN model was trained using the Planet and SPOT data simultaneously. To evaluate the model’s performance, three test sets were considered: only the Planet test images, only the SPOT test images, and both the Planet and SPOT images. The images for Planet and Spot covered the same territory.

### 2.5. Training Setup

The training of all neural network models was performed on a PC with GTX-1080Ti GPUs, using Keras [47] with a Tensorflow [48] backend. For the simple regression model, the following training parameters were set. An optimizer RMSprop was chosen with a learning rate of 0.001, which was reduced with patience 5. There were 20 epochs with 100 steps per epoch. The batch size was specified to be 30 with an image size of 256×256 pixels [24]. A model based on GAN training parameters was constructed as follows. The loss functions were chosen to be binary cross-entropy and MAE. The optimizer was Adam. The batch size and image size were the same as for the simple model. The models were trained for 600 epochs, 100 steps per epoch, and a batch size of 30. For the Planet data, we also conducted a fine-tuning procedure of the pretrained generative model using a small area without the necessity of markup. For the SPOT data, there was no additional training.

## 3. Results and Discussion

The results for NIR generation by cGAN are presented in Table 1 for the WorldView, SPOT, and Planet satellite data. All values for real and generated NIR were in the range [0,1]. The simple CNN regression approach showed significantly poor results (the MAE was 0.21 for WorldView). Therefore, we did not select this approach for future study. The principal difference between cGANs and the regression CNN model is the type of loss function. As our experiments show, both MAE and MSE loss in the regression CNN model led to the local optimum, which was far from the global one. The loss function can be affected by the distribution of RGB values. Compared to the regression CNN model, the results of cGAN were significantly closer to the real NIR values.

**Figure 5:**
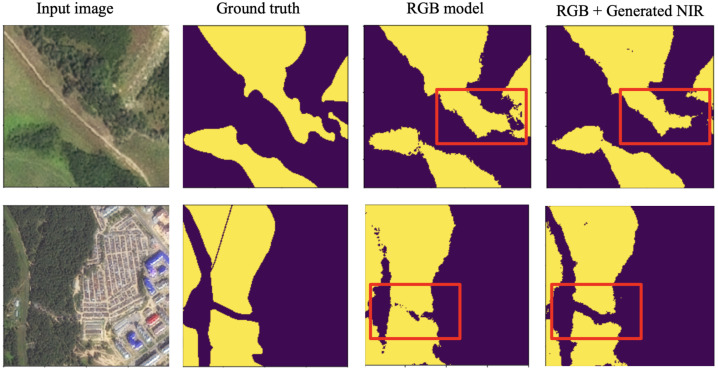
Forest segmentation predictions on the test regions (SPOT). One model was trained just on RGB images; another model used RGB and generated NIR.

**Figure 6:**
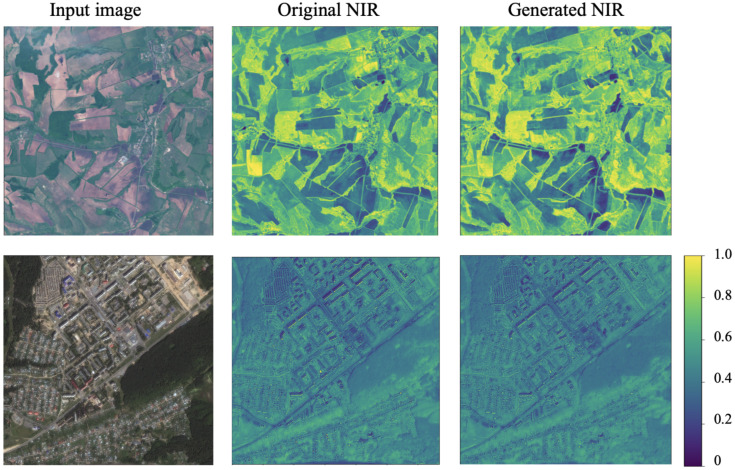
Example of generated NIR on the test set. The first row presents the SPOT image; the second row is the WorldView image.

Another approach to evaluate the generated NIR band involves the forest segmentation task. The segmentation model was trained on the original NIR channels to predict the forest segmentation mask using RGB & generated NIR. The results are presented in Table 2, which shows that the additional NIR channel improved the cross-domain stability of the model. The example of segmentation prediction is shown in Figure 5. The model using the generated NIR provided more accurate results than the model trained only on RGB bands. The original NIR usage obtained an F1-score of 0.953, the generated NIR obtained an F1-score of 0.947, and the model using only RGB bands obtained an F1-score of 0.914. The predicted NIR channel is shown in Figure 6, which confirms a high level of similarity between generated and original bands. Therefore, this approach allows more efficient CNN model usage in practical cases when data from different Basemaps are processed and cross-domain tasks occur.

We also assessed the generated and original NIR bands using classical machine learning approach. Results are presented in Table 2. For RF, the NIR band usage improves the classification quality from 0.841 to 0.877. The F1-score for the generated NIR is 0.874. This experiment shows that for the classification approach without spatial information the generated band is also provide significant information.

The results for different dataset sizes are presented in Table 3 and show that leveraging the NIR channel was beneficial in the case of smaller dataset sizes, whereas its effect decreased with the growing amount of the training data.

GANs aim to learn dataset distribution. It is conducted by minimizing the overall distance between the real and the generated distribution. We made an assumption that the dataset size is enough to approximate the distribution. Thus, we train the generator to sample according to the target distribution. The trained generator allowed a high-realistic image-to-image translation (G: {RGB} → NIR) such that the obtained NIR band is similar to those belonging to the target domain.

**Figure 7:**
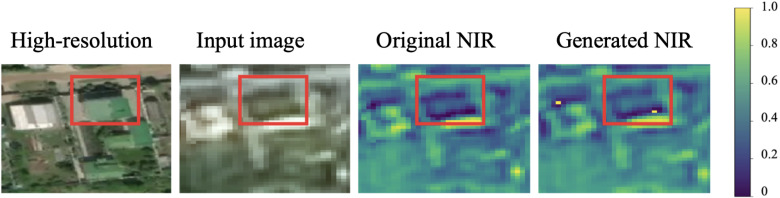
Example of a case with a green roof (SPOT image). The green roof has low NIR values both for original and generated NIR bands.

Example of a green roof is presented in Figure 7. Although, the color of the object is green, NIR value is low. It shows that the model had a sufficient amount of the training samples to learn such cases.

The experiments indicate that the generated NIR provides additional information to the segmentation model. We assume that the generative model incorporates the hidden statistical connections between the spectral channels that can be learned from the significant amount of real RGB and NIR data. As opposed to the segmentation or classification approach, the channel generation does not require the manual ground truth markup to significantly increase the dataset size. Therefore, this approach can be used as a feature-engineering tool to create a new feature similar to the NIR band of multispectral remote sensing imagery.

We set the goal to predict exactly the NIR band instead of vegetation indexes such as NDVI or EVI. These indexes use the NIR band in combination with the Red band. The NIR band generation allows further computation of other indexes without a requirement for extra model training. The future study can be extended by implementing different vegetation indexes. Moreover, in the case of using neural networks with the generated NIR band, it is enough to provide input NIR and Red bands separately (not in the form of the computed indexes) because a neural network can approximate nonlinear functions such as vegetation indexes.

**Figure 8:**
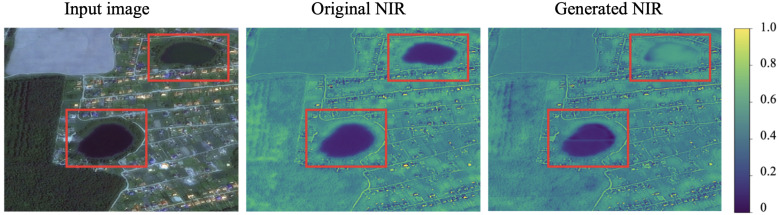
Example of a failure case (SPOT image). Green lake is erroneously treated as a surface with high NIR value.

One example of a failure case is a green lake (Figure 8) that might be mistaken for a green lawn. The reason is insufficient representation in the training dataset. Another possible example is an artificial turf such as an open-air stadium. The model can erroneously treat it as a landcover with high NIR value. On the other hand, if we add a significant amount of such samples, it is possible that the model learns such a distribution both.

Pix2pix architecture includes 54M parameters in the generator part and 6M parameters in the discriminator part. The future study can be focused on trainable parameters reduction. Light-weighted neural network models are studied currently and show promising results in the remote sensing domain [34] (just 4M parameters are used).

Training models independently for each data source often leads to better results. However, it is a more expensive approach. In this study, we considered the case when we minimize the cost. In future research, separate models training for each datasource should be studied and analyzed.

Data providers aim to minimize time and other costs while providing imagery to customers. For this purpose, online services for data acquisition are created [49,50] that allows one to analyze data “on the fly”. The most spread and cheap format for such platforms is RGB images, even when original imagery includes more spectral channels. The proposed NIR generation approach can be implemented for such products as “basemaps”. That requires further study.

In the future, we seek to implement this feature-engineering approach to other remote sensing tasks, such as agriculture classification and land-cover semantic segmentation. In addition, the proposed approach holds potential to solve challenges when only drones’ RGB channels are available. Another direction is to combine this feature-engineering approach with different augmentation techniques for remote sensing tasks [51,52].

It is promising to investigate the application of NIR generation methods beyond remote sensing problems in future works. Since NIR provides valuable auxiliary data in plant phenotyping tasks, NIR generation can be extended for greenhouses where high precision is vital [53].

## 4. Conclusions

The NIR band contains essential properties for landcover tasks. However, in particular cases, this band is not available. This study investigated Pix2pix cGAN implementation for image-to-image translation from RGB space imagery to the NIR band. We proposed an efficient feature-engineering approach based on an artificial NIR band generation. We conducted forest segmentation experiments to assess the importance of the NIR band in cases of small datasets and different satellite data sources. The proposed approach improved the model’s robustness to data source diversity and reduced the requirement to mark the dataset size, which is crucial for machine learning challenges. We assume that this data generation strategy can be implemented in practical tasks that require the NIR channel. This method can be extended to other spectral channels and remote sensing data sources.

## Figures and Tables

**Table 1:** Error of the artificial NIR band for the test WorldView, SPOT, and Planet imagery.

	MAE	RMSE	Mean Bias
WorldView	0.09	0.31	0.058
SPOT	0.037	0.194	−0.0029
Planet	0.16	0.41	0.088

**Table 2:** The results of the forest segmentation experiments with different data sources. Both the RGB model and the RGB and NIR model were trained on Planet and Spot images simultaneously. The F1-score was computed on the test set individually for Planet and Spot and for the joined Planet and Spot test set.

	U-Net			RF		
Test images	RGB	RGB	RGB and	RGB	RGB	RGB and
		and NIR	artificial NIR		and NIR	artificial NIR
SPOT	0.954	0.961	0.96	0.874	0.892	0.889
Planet	0.857	0.939	0.936	0.815	0.863	0.861
SPOT + Planet	0.932	0.96	0.945	0.836	0.876	0.872
Average	0.914	0.953	0.947	0.841	0.877	0.874
		(+0.039)	(+0.033)		(+0.036)	(+0.033)

**Table 3:** The results for the forest segmentation experiments with different dataset sizes. The F1-score for SPOT and Planet on the test set. The entire data size was 500,000 ha.

	Bands	All Data	1/2	1/3
SPOT	RGB	0.97	0.956	0.942
	RGB and NIR	0.97	0.963	0.961
Planet	RGB	0.939	0.933	0.874
	RGB and NIR	0.95	0.942	0.927

## Data Availability

Not applicable.
